# Inhibition of Colony Stimulating Factor 1 Receptor Suppresses Neuroinflammation and Neonatal Hypoxic-Ischemic Brain Injury

**DOI:** 10.3389/fneur.2021.607370

**Published:** 2021-02-18

**Authors:** Bohao Zhang, Yunwei Ran, Siting Wu, Fang Zhang, Huachen Huang, Changlian Zhu, Shusheng Zhang, Xiaoan Zhang

**Affiliations:** ^1^Henan Key Laboratory of Child Brain Injury, Institute of Neuroscience and Third Affiliated Hospital of Zhengzhou University, Zhengzhou, China; ^2^Center of Advanced Analysis & Gene Sequencing, Zhengzhou University, Zhengzhou, China; ^3^Medical Research Center, The Third Affiliated Hospital of Zhengzhou University, Zhengzhou, China; ^4^Department of Neurology, Tianjin Neurological Institute, Tianjin Medical University General Hospital, Tianjin, China; ^5^Department of Clinical Neuroscience, Center for Brain Repair and Rehabilitation, Institute of Neuroscience and Physiology, University of Gothenburg, Gothenburg, Sweden

**Keywords:** colony stimulating factor 1 receptor, PLX3397, microglia, neuroinflammation, neonatal hypoxic-ischemic brain injury

## Abstract

Hypoxic-ischemic (HI) brain injury is a major cause of neonatal death or lifetime disability without widely accepted effective pharmacological treatments. It has been shown that the survival of microglia requires colony-stimulating factor 1 receptor (CSF1R) signaling and microglia participate in neonatal HI brain injury. We therefore hypothesize that microglia depletion during a HI insult period could reduce immature brain injury. In this study, CD1 mouse pups were treated with a CSF1R inhibitor (PLX3397, 25 mg/kg/daily) or a vehicle from postnatal day 4 to day 11 (P4–11), and over 90% of total brain microglia were deleted at P9. Unilateral hemisphere HI injury was induced at P9 by permanently ligating the left common carotid arteries and exposing the pups to 10% oxygen for 30 min to produce moderate left hemisphere injury. We found that the PLX3397 treatment reduced HI brain injury by 46.4%, as evaluated by the percentage of brain infarction at 48 h after HI. Furthermore, CSF1R inhibition suppressed the infiltration of neutrophils (69.7% reduction, *p* = 0.038), macrophages (77.4% reduction, *p* = 0.009), and T cells (72.9% reduction, *p* = 0.008) to the brain, the production of cytokines and chemokines (such as CCL12, CCL6, CCL21, CCL22, CCL19, IL7, CD14, and WISP-1), and reduced neuronal apoptosis as indicated by active caspase-3 labeled cells at 48 h after HI (615.20 ± 156.84/mm^2^ vs. 1,205.00 ± 99.15/mm^2^, *p* = 0.013). Our results suggest that CSF1R inhibition suppresses neuroinflammation and neonatal brain injury after acute cerebral hypoxia-ischemia in neonatal mice.

## Introduction

Perinatal hypoxic-ischemic (HI) brain injury is a clinical syndrome associated with oxygen deprivation and is a major cause of neonatal death and long-term neurofunctional disabilities with limited therapies available (1–7). HI causes additional oxidative stress, excitotoxicity, and neuroinflammation, which further exacerbate the initial brain injury (2, 7). Neuroinflammation occurs as early as minutes after the insult and is a key contributor to acute perinatal HI brain injury (8–11). Brain resident microglia play essential roles in immune surveillance of the brain microenvironment. After cerebral HI, brain microglia change their phenotype, promote the production of inflammatory factors, and recruit peripheral immune cells (12–14). The activated microglia change to the anti-inflammatory state and promote angiogenesis, tissue remodeling, and neurorepair at later stages after HI brain injury (14). However, the role of microglia in perinatal HI brain injury remains poorly understood (14).

Colony-stimulating factor 1 receptor (CSF1R) signaling is essential for microglia survival. Pexidartinib (PLX3397) is a CSF1R antagonist that has been reported to eliminate brain microglia temporarily and recoverably in adult mice (15–17). However, whether CSF1R inhibition can effectively eliminate microglia in the neonatal brain remains unknown. In this study, we determined the effects of CSF1R inhibition on microglia in the neonatal brain and cerebral HI injury using a mouse model.

## Materials and Methods

### Animals

CD1 pregnant mice and their neonatal pups were used in this study. CD1 pregnant mice were purchased from Beijing Huafukang Biotechnology Co., Ltd. (Beijing, China). The mice were housed under controlled conditions, with free access to food and water, at 23 ± 2°C, with 12 h light/dark periods. All the experiments were approved by the Committee on the Ethics of Animal Experiments of Tianjin Neurological Institute (Tianjin, China).

### Administration of PLX3397

PLX3397 (Selleckchem, Houston, TX, USA) was dissolved in dimethyl sulfoxide as previously described, to a final concentration of 50 mg/ml (16, 18). At postnatal day 4 (P4), both the male and female littermate pups were randomly divided into two groups. One group was orally administered PLX3397 (25 mg/kg) twice a day starting from P4 to the end of the experiment (P11), and the control group was given the same volume of vehicle. With this administration procedure of PLX3397, over 90% of total brain microglia were deleted at P9 or P11 in mice when tested by cell analysis with flow cytometry. Moreover, no serious side effects such as death or body weight loss were found in mice treated with PLX3397 in this study.

### Hypoxic-Ischemic Model

P9 male and female CD1 mouse pups of 5–7 g bodyweight were used. A moderate cerebral HI brain injury were induced by permanent ligation of the left common carotid artery combined with exposure to 10% oxygen for 30 min as previously described (19, 20). In brief, mouse pups were anesthetized with isoflurane (3% for induction and 1.5% for maintenance during surgery). A midline cervical incision was made in the anterior neck. The left common carotid artery was isolated and ligated with a sterile surgical line to occlude blood flow from P9 to P11. After surgery, the pups were placed back with the dams for 1 h. The pups were then exposed to hypoxic conditions (10% O_2_ and 90% N_2_, 37°C) for 30 min. Following exposure, the pups were returned to the cages with the dams and were reared for another 48 h prior to assessment of brain injury and inflammation. This model is widely accepted as mimicking neonatal asphyxia and is known as the Rice–Vannucci model. The brain lesion can be found in the cortex, hippocampus, striatum, and thalamus, similar to the clinical finding in neonatal asphyxia (21). In this model, only the combination of unilateral permanent ligation and hypoxia can induce brain injury in the ipsilateral hemisphere. The hypoxia time needed adjustment to produce moderate brain injury because of the variable sensitivity to hypoxia of species, genetic background, and age of the mouse pups (22, 23). To maintain a consistent HI effect in mice, we strictly followed the experimental procedures and conditions in different batches of experiments. Each batch of HI induction was carried out in parallel for mice treated with PLX3397 and the vehicle.

### Corner Turn Test

The corner-turning test was conducted to evaluate unilateral abnormalities of sensory and motor functions of HI mice. At 48 h after HI induction, mouse pups were put to a small opening side of a 30° angle corner formed by two boards and encouraged to go into the corner. The mouse had to turn right or left to leave the corner. Each mouse repeated this procedure 10 times with an interval of ≥ 30 s between trials. The percentage of ipsilateral turns was then calculated as ipsilateral turns/(ipsilateral turn + contralateral turn) × 100.

### 2,3,5-Tripenyltetrazolium Chloride (TTC) Staining

At 48 h after HI, the pups were deeply anesthetized with isoflurane. The brain was immediately isolated after perfusion with 10 ml ice-cold phosphate buffered saline (PBS) and stored at −20°C for 20 min and subsequently was sliced into 1.5 mm coronal sections. The sections were immersed in 2% TTC solution (Sigma-Aldrich, USA) at 37°C for 20 min in the dark. After washing with distilled H_2_O, the sections were fixed in 4% paraformaldehyde for 20 min and photographed. The infarcted portions were identified as the white areas without TTC staining. The infarcted areas of each section were traced and measured using Image-Pro Plus 6.0 (U.S. National Institutes of Health, Washington, DC) (24). The total infarct volume was calculated by summation of the representative infarct volume of each section. The infarction percentage was calculated as follows: ([total contralateral hemispheric volume] – [total ipsilateral hemispheric stained volume]) / (total contralateral hemispheric volume × 2) × 100% (19, 25).

### Flow Cytometry

Flow cytometry was performed to analyze the number of microglia and infiltration of immune cells in the brain. The pups were anesthetized and perfused intracardially with ice-cold 0.1 M PBS (pH 7.4). The brain was then isolated and rapidly placed in ice-cold PBS. The olfactory bulb, cerebellum, and brain stem were discarded, and only the cerebral hemispheres were retained. The cerebral tissue was cut with scissors and mechanically passed through a 40 μm filter to obtain a cell suspension. To remove the myelin sheath from the cell suspension, 10 ml of 30% Percoll (Sigma, St. Louis, MO) was added to the brain tissue suspension, followed by density gradient centrifugation at 4°C, 600 g for 20 min. The isolated cell suspensions were stained with fluorescent-dye conjugated anti-mouse antibodies: CD45 (103108, BioLegend, San Diego, CA), CD11b (101216, BioLegend, San Diego, CA), Ly6G (127608, BioLegend, San Diego, CA), F4/80 (123116, BioLegend, San Diego, CA), CD3 (100327, BioLegend, San Diego, CA), CD19 (115519, BioLegend, San Diego, CA), and CSF1R (135510, BioLegend, San Diego, CA, USA). A FACS Aria III flow cytometer (BD Biosciences, San Jose, CA) was used to acquire flow cytometric data. Data were analyzed using Flow Jo software, version 10.0.7 (Informer Technologies, Walnut, CA).

### Immunofluorescence Staining

The animals were anesthetized and perfused intracardially with ice-cold 0.1M PBS (pH 7.4) followed by 4% paraformaldehyde. The cerebrums were post-fixed in 4% paraformaldehyde for 48 h, embedded in paraffin, and cut into 5 μm coronal sections. The paraffin-embedded sections were heated at 95°C in 0.01 M sodium citrate buffer solution (pH 6.0) for 30 min to repair the antigen. The sections were blocked in 5% bovine serum albumin in PBS containing 0.2% Triton at 25°C for 2 h, and then incubated with mouse anti-mouse NeuN (ab104224, Abcam, 1:1000 dilution) and rabbit anti-mouse caspase-3 (Cell Signaling Technology, 9661S; 1:1000) as primary antibodies at 4°C overnight. After washing thrice with PBS, the sections were incubated with biotinylated goat anti-mouse 488 (A11001, Invitrogen, Carlsbad, CA, 1:500 dilution) and goat anti-rabbit 546 (A11010, Invitrogen, Carlsbad, CA, 1:500 dilution) secondary antibodies at room temperature for 1 h. The stained sections were imaged with a fluorescence microscope (Model BX-61, Olympus, Center Valley, PA, USA). Sections without antibody staining were used as negative controls.

### Real-Time PCR

Total RNA was isolated using TRIzol reagent (Thermo Fisher Scientific). Analysis was performed in a thermocycler (7500, Applied Biosystems, USA) using the following conditions: initial denaturation for 10 s at 95°C, 40 cycles of 5 s denaturation at 95°C, 30 s annealing at 60°C, and 1 min extension at 95°C. The following primers were used for analysis: CCL6 forward GGCTTTCAAGACACTTCTTCAG, CCL6 reverse CCCTCCTGCTGATAAAGATGAT; CCL12 forward CCAGTCACGTGCTGTTATAATG, CCL12 reverse AGACGTCTTATCCAAGTGGTTT; CD14 forward CAAGTTCCCGACCCTCCAAG, CD14 reverse GCA- TCCCGCAGTGAATTGTG; CX3CL1 forward GAGGCCACCCTAGACCACTA, CX3CL1 reverse CACTGGAGTTGGGGTGTCAA; M-CSF forward TGATTGGGAATGGACACCTG, M-CSF reverse AAAGGCAATCTGGCATGAAGT; IL-7 forward TCTGCTGCCTGTCACATCATCT, IL-7 reverse AAGTTTGGTTCATTATTCGGG; ICAM-1 forward TGTCAGCCACCATGCCTTAG, ICAM-1 reverse CAGCTTGCACGACCCTTCTA; VEGF forward GCCAGACAGGGTTGCCATAC, VEGF reverse GGAGTGGGATGGATGATGTCAG; WISP-1 forward AACTGCATAGCCTACACTAGTC, WISP-1 reverse ATTGACGTTAGAGATCCGAGTG; and β-actin forward CTACCTCATGAAGATCCTGACC, β-actin reverse CACAGCTTCTCTTTGATGTCAC. The relative expression levels were calculated using the 2^−ΔΔCt^ method and normalized with β-actin as the internal reference.

### Cytokine Array

Cytokines in the cerebral tissue lysates were measured using a mouse XL cytokine array kit (ARY028, R&D Systems). Briefly, cerebral tissue was removed and homogenized in PBS with protease inhibitors, aprotinin (A6279, Sigma), leupeptin (1167, Tocris), and pepstatin (1190, Tocris). After homogenization, Triton X-100 was added to the samples to a final concentration of 1%. Protein homogenates were centrifuged at 10,000 × g for 5 min to remove cellular debris and stored at −80°C until assay. The protein levels of cytokines were detected by exposure of the membrane to the X-ray film. The Gel-Pro analyzer was used to evaluate the intensity of the developed dots.

### Statistical Analysis

All statistical data are presented as mean ± SEM. Experimental and control group allocations, data collection, and data analysis were blinded by using different investigators or masking sample labels. Animals were randomly assigned to experimental groups. All experiments were successfully reproduced at least three times. A two-tailed unpaired Student's *t*-test was used to compare the two groups. One-way ANOVA was used for comparison between two groups. Two-way ANOVA with multiple comparisons was used to compare multi-group data. A level of *p* < 0.05 was considered significant. Statistical analyses were performed using the Prism 6.0 software (GraphPad).

## Results

### PLX3397 Reduces Brain Microglia Numbers and Brain Injury in Neonatal Mice

**Figure 1:**
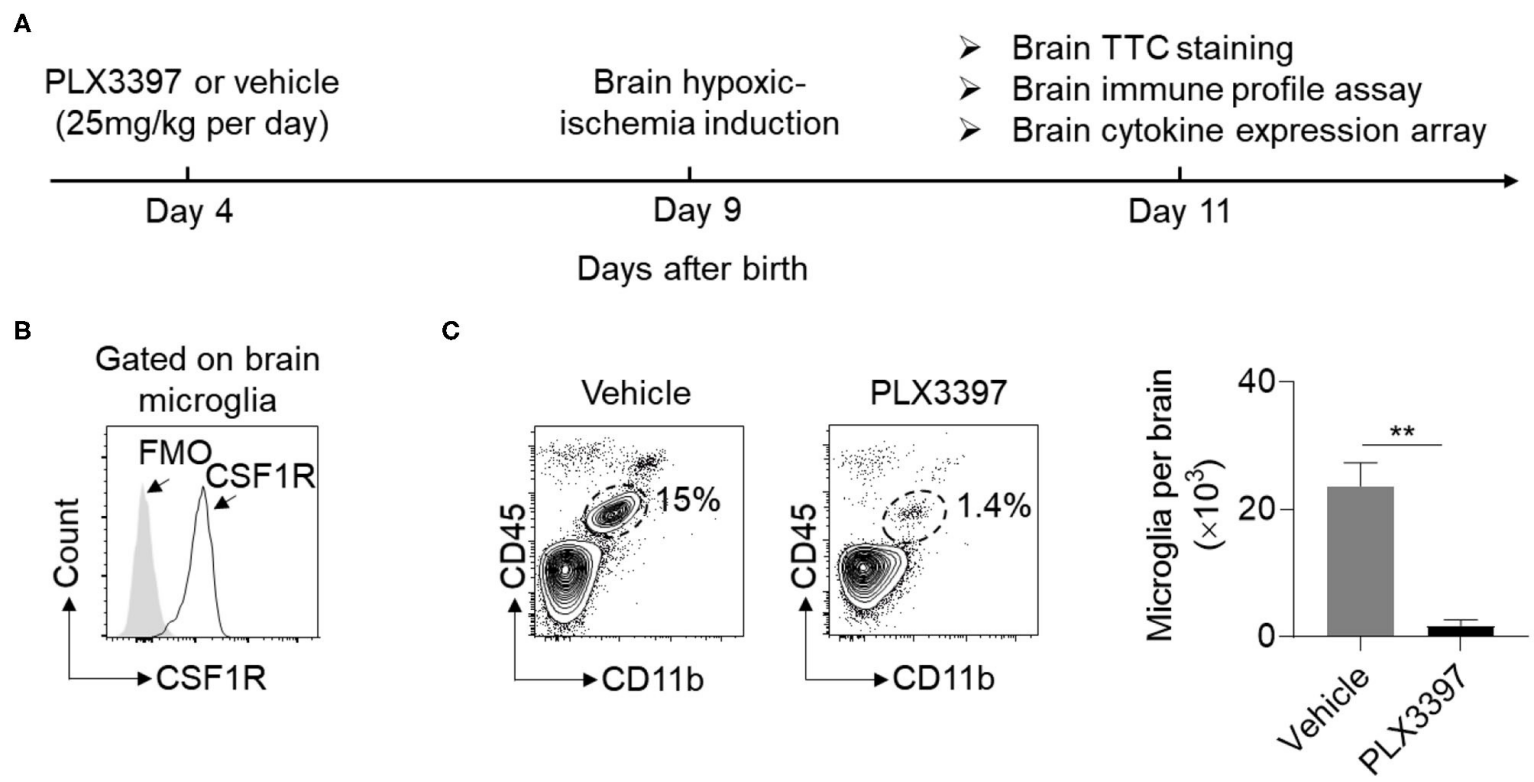
PLX3397 reduces brain microglia in neonatal CD1 mice. **(A)** Experimental schematic design of PLX3397 administration in neonatal CD1 mice. **(B)** Expression of CSF1R on brain microglia (CD45^int^ CD11b^+^ cells) of neonatal CD1 mice at 4 days after birth. FMO stands for Fluorescence Minus One Control. **(C)** Flow cytometry plots and statistics of brain microglia (CD11b^+^CD45^int^ cells) at 9 days after birth in neonatal CD1 mice treated with PLX3397 (*n* = 6, 3 males and 3 females) or the vehicle (*n* = 6, 3 males and 3 females). Unpaired two-tailed *t*-test. Data are presented as mean ± SEM, **p < 0.01.

To deplete brain microglia, neonatal mice were treated with PLX3397 (25 mg/kg body weight) or vehicle at P4 prior to HI induction (Figure 1A). We found that brain microglia expressed CSF1R in neonatal mice (Figure 1B), and oral administration of PLX3397 for five consecutive days can effectively reduce ~90% of microglia in the brain of normal neonatal mice (Figure 1C).

**Figure 2:**
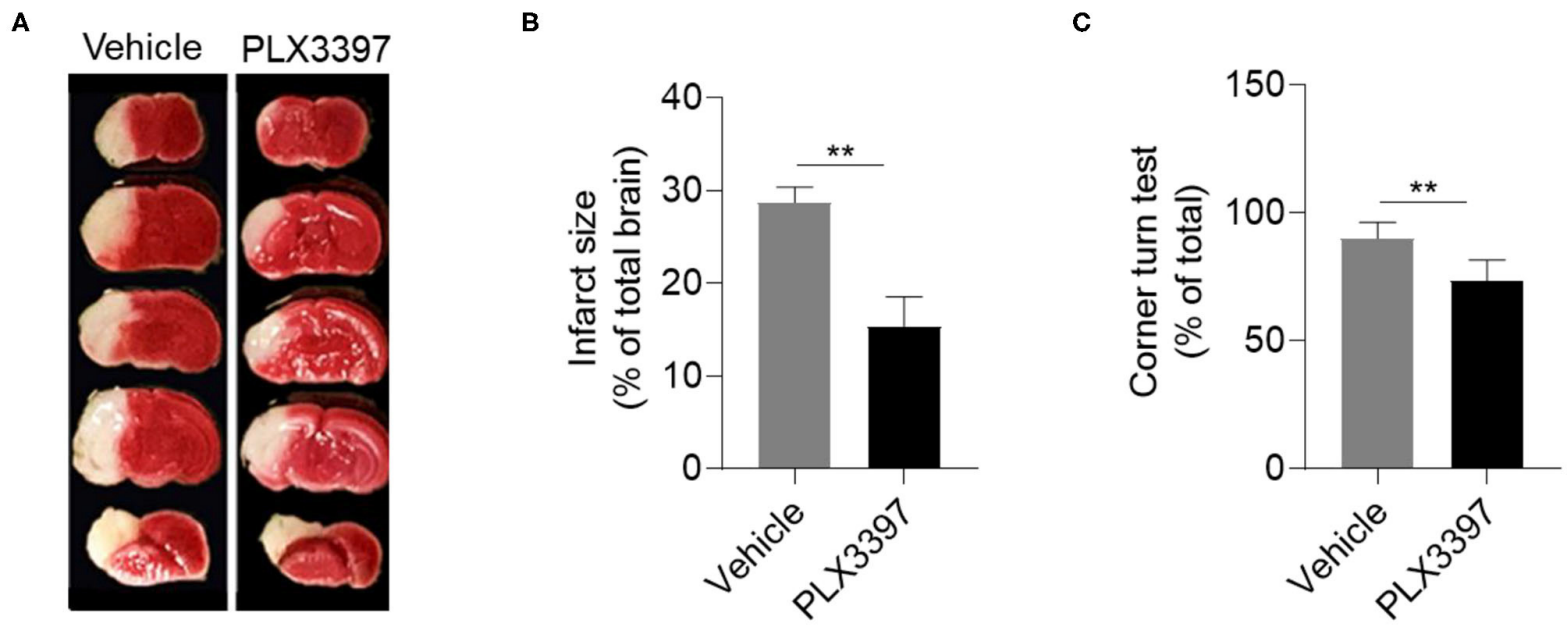
PLX3397 attenuates neonatal hypoxic-ischemic brain injury in mice. **(A)** 2,3,5-Tripenyltetrazolium chloride (TTC) staining shows brain injury at 48 h after hypoxic-ischemic brain injury induction in neonatal CD1 mice treated with PLX3397 or the vehicle. **(B)** Statistics of infarct size measured by TTC staining in HI mice treated with PLX3397 (*n* = 9, 6 males and 3 females) or vehicle (*n* = 11, 5 males and 6 females) Unpaired two-tailed *t*-test. **(C)** Corner turn test at 48 h after HI induction in mice treated with PLX3397 (*n* = 6, 3 males and 3 females) or vehicle (*n* = 6, 3 males and 3 females). Unpaired two-tailed *t*-test. Data are presented as mean ± SEM, **p < 0.01.

Induction of HI in neonatal CD1 mice was performed at P9. We then evaluated HI-induced brain lesion size using TTC staining. Compared to HI mice treated with the vehicle, brain infarct size was significantly decreased in the mice treated with PLX3397 at 48 h after HI (Figure 2A). The relative infarct size was 28.70 ± 1.66% in HI mice treated with the vehicle and 15.37 ± 3.17% in HI mice treated with PLX3397, which accounted for a 46.4% reduction of brain injury (*p* = 0.001) (Figure 2B). We further analyzed the infarction by gender and the relative infarct size was 27.3 ± 3.4% in males and 29.8 ± 1.4% in females in the vehicle treated group (*p* = 0.105, data not shown), and 15.3 ± 4.9% in males and 15.4 ± 0.4% in females in the PLX3397 treated group (*p* = 0.99, data not shown). These results indicate that inhibition of CSF1R prevents brain injury in neonatal HI mice and there was no gender difference in brain injury in either vehicle of PLX3397 treated mice at 48 h after HI induction.

As unilateral visible lesions were found after HI induction (Figure 2A), we further used the corner turn test to analyze unilateral disability in motor and sensory function of HI mice as previous published (26). At 48 h after HI induction, our data indicated that HI mice treated with PLX3397 had less unilateral asymmetry than HI mice treated with the vehicle (90 ± 2.6% vs. 73.4 ± 3.3%, *p* = 0.0087) (Figure 2C).

### CSF1R Inhibition Reduces the Recruitment of Circulating Immune Cells Into the Brain

**Figure 3:**
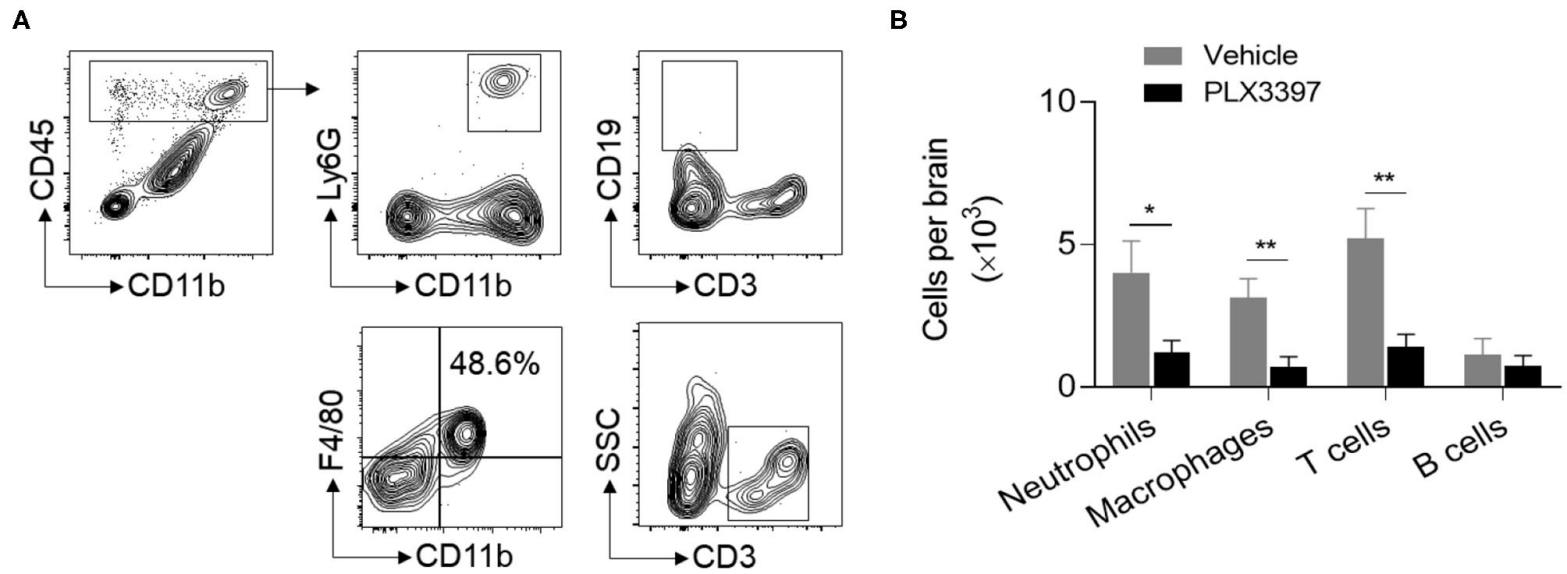
PLX3397 reduces immune cell recruitment into the brains of HI mice. **(A)** Flow cytometry gating strategy for neutrophils (CD45^high^CD11b^+^Ly6G^+^), macrophages (CD45^high^CD11b^+^F4/80^+^), T cells (CD45^high^CD3^+^), and B cells (CD45^high^CD3^−^CD19^+^) in the brains of neonatal HI mice. **(B)** The quantitation of brain neutrophils, macrophages, T cells, and B cells at 48 h after HI in mice treated with PLX3397 (*n* = 6, 4 males and 2 females) or the vehicle (*n* = 6, 4 males and 2 females). Unpaired two-tailed *t*-test. Data are presented as mean ± SEM, *p < 0.05, **p < 0.01.

In addition to microglia, brain-recruited neutrophils, macrophages, and lymphocytes also contribute to brain injury in HI mice. Thus, we measured whether PLX3397 affects brain infiltration of peripheral immune cells in neonatal HI mice. At 48 h after HI induction, our data indicated that the number of neutrophils, macrophages, and T cells in the whole brain tissue was much lower in mice treated with PLX3397 (Figure 3). The number of neutrophils, macrophages, and T cells were 4.02 ± 1.10 × 10^3^, 3.14 ± 0.66 × 10^3^, and 5.21 ± 1.06 × 10^3^ in HI mice treated with the vehicle and 1.22 ± 0.40 × 10^3^, 0.71 ± 0.36 × 10^3^, and 1.41 ± 0.45 × 10^3^ in HI mice treated with PLX3397, which accounted for a 69.7, 77.4, and 72.9% reduction of infiltration of neutrophils (*p* = 0.038), macrophages (*p* = 0.009), and T cells (*p* = 0.008) to the brain. This result suggests that inhibition of CSF1R by PLX3397 attenuates neuroinflammation in HI mice.

### CSF1R Inhibition Alters Cytokine/Chemokine Profile in the Mouse Brain After HI Injury

**Figure 4:**
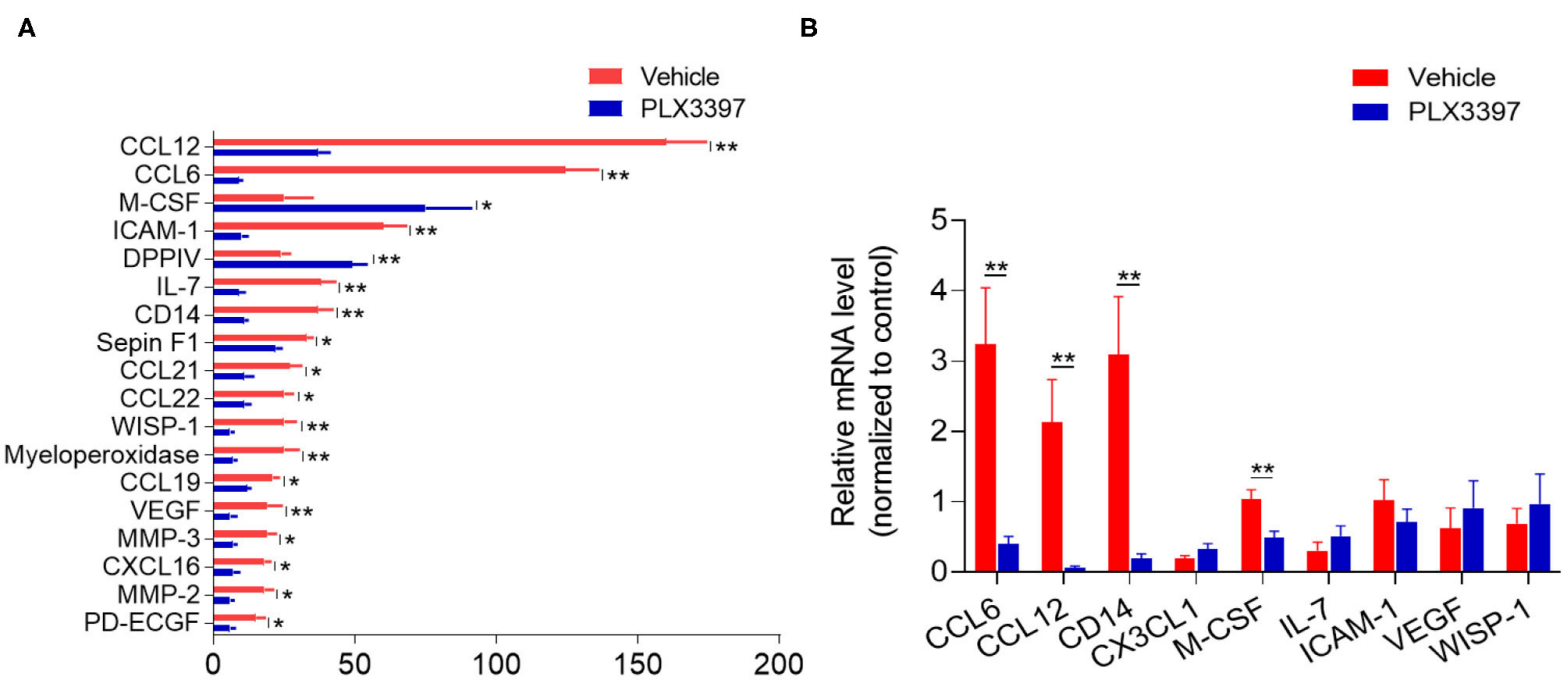
PLX3397 changes brain cytokine/chemokine profile in HI mice. **(A)** The total differentially expressed cytokines/chemokines in the brains of HI mice treated with PLX3397 or the vehicle. Brain tissues were collected at 48 h after HI induction and the protein levels were measured with a cytokine/chemokine profile ELISA array kit in which 111 factors were detected. *n* = 3 independent experiments. Each independent experiment needed 10 mice per group (5 males and 5 females). Unpaired two-tailed *t*-test. **(B)** The mRNA levels of representative differentially expressed factors related to regulation of chemotaxis (CCL2, CCL12), immune response (CD14, M-CSF, IL-7), and BBB integrity (ICAM-1, VEGF, and WISP-1). CX3CL1 was as a negative control that was not significantly changed in protein level between HI mice treated with PLX3397 or the vehicle (data not shown). Brain tissues were collected at 48 h after HI from HI mice treated with PLX3397 or the vehicle. *n* = 5 mice per group. Unpaired two-tailed *t*-test. Data are presented as mean ± SEM, *p < 0.05, **p < 0.01.

Chemokines and cytokines are important soluble factors in regulating the immune response triggered by tissue injury. As PLX3397 treatment reduced immune cells in injured brain tissue of HI mice (Figure 3), we wondered whether there is any difference in the cytokine/chemokine expressing profile between HI mice treated with PLX3397 and the vehicle. We used a proteome profiler mouse XL cytokine array to assess the expression of hundreds of cytokines/chemokines in the brain lysate of HI mice treated with PLX3397 or the vehicle. In Figure 4A, we show the 18 differently expressed factors at protein level between HI mice treated with PLX3397 or the vehicle (*p* < 0.05). In these differently expressed factors except M-CSF and DPPIV, their protein levels were reduced in the brains of HI mice treated with PLX3397. Fifteen of these 18 factors were related to chemotaxis and immune response (e.g., CCL6, CCL12, CCL19, CCL21 CCL22, CXCL16, CD14, IL-7, M-CSF, and myeloperoxidase) or the regulation of blood-brain barrier integrity (MMP-2, MMP3, ICAM-1, VEGF, and WISP-1).

We further analyzed the brain mRNA levels of some representative differently expressed factors related to regulation of chemotaxis (CCL2, CCL12), immune response (CD14, M-CSF, IL-7), and blood brain barrier (BBB) integrity (ICAM-1, VEGF, WISP-1). However, our data indicated that not all these representative factors had synergistic changes in both mRNA and protein level in the brain of HI mice treated with PLX3397 (e.g., IL-7, ICAM-1, VEGF, and WISP-1) (Figure 4B). CX3CL1 was used as a negative control that is not significantly changed at protein level in the brain by cytokine array (data not shown), and we also did not find a difference in mRNA level of brain CX3CL1 between HI mice treated with PLX3397 and the vehicle (Figure 4B). These results demonstrated that PLX3397 alters the transcription or (and) translation of some factors in regulating immune cells' mobilization and BBB integrity in the brain after HI injury.

### CSF1R Inhibition Attenuates Neuronal Death in Injured Brain Tissue in HI Mice

**Figure 5:**
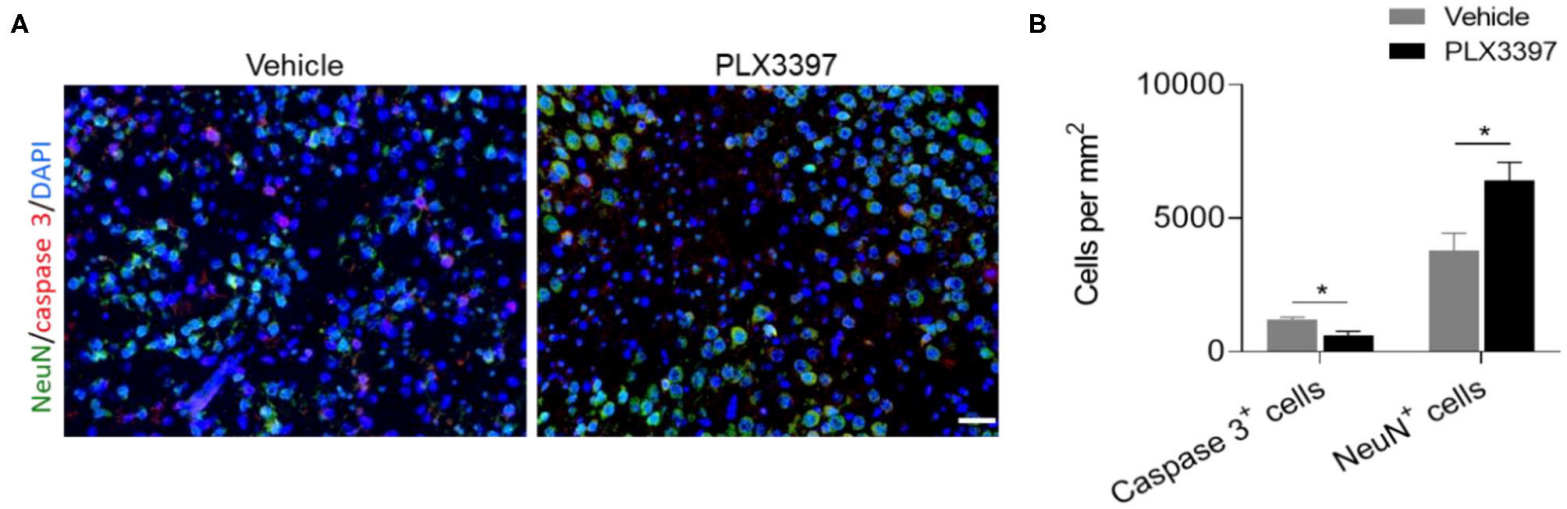
PLX3397 attenuates cell death in injured brain tissue of HI mice. **(A)** immunostaining of activated caspase 3 (red) and neuron (green) in injured brain tissue at 48 h after HI induction in mice treated with PLX3397 or the vehicle. Scale bar = 20 μm. **(B)** Quantification of neuron and cell apoptosis at 48 h after HI induction in injured brain tissue of HI mice treated with PLX3397 (*n* = 6, 1 male and 5 females) or vehicle (*n* = 6, 3 males and 3 females). Unpaired two-tailed *t*-test. Data are presented as mean ± SEM, *p < 0.05.

To determine the effects of PLX3397 on HI-induced neuronal death, we quantified the numbers of NueN^+^ cells and active caspase 3^+^ cells in the lesion area of HI mice at 48 h after HI induction. Compared to mice treated with the vehicle, the PLX3397 treatment preserved more neurons (6,423.60 ± 681.18 vs. 3,790.40 ± 656.80, *p* = 0.024) in injured brain tissue (around the infarct in the cortex) of HI mice, accompanied by a reduction in cell apoptosis (615.20 ± 156.84 vs. 1,205.00 ± 99.15, *p* = 0.013) (Figure 5). These results indicate that inhibition of CSF1R by PLX3397 reduces cellular apoptosis in injured brain tissue in HI mice.

## Discussion

In the present study, we showed that CSF1R inhibition reduces brain injury in neonatal mice after HI insult. As documented here, most microglia can be effectively eliminated by CSF1R inhibition in neonatal mice. In contrast to adult mice that require 2–3 weeks of 50 mg/kg PLX3397 treatment to eliminate 90% of brain microglia (15), we found that a lower dosage of PLX3397 (25 mg/kg) for 5 days can effectively reduce ~90% of microglia in the neonatal mouse brain. Importantly, the reduced neuronal death and infarct size suggest that targeting CSF1R could be a promising approach to limiting HI injury in the neonatal brain.

Our study provides new evidence that microglia depletion reduces brain ischemia injury in mice, which is opposite to some previous studies with brain ischemic injury models (15, 27). This discrepancy could be related to age, gender, strain of animals, and the type of models used (27–31). With different strains, age, and gender of mice, there are controversial results on whether microglia are protective or harmful to ischemic brain injury (14, 15, 32–35). For example, microglia depletion with PLX3397 provided neuroprotective effects by an inhibitory action on the astrocyte response in adult transient middle cerebral artery occlusion mice (15). However, the role of astrocytes in the development of ischemic brain lesions is unclear in neonates (14). Meanwhile, neonatal and adult microglia have a distinguishing immune response and cytokine/chemokine production profile after encountering brain ischemia. For example, adult microglia are more sensitive to the stimulation of Toll-like receptors ligands and produce higher matrix metalloproteinases, while the ability to respond to nitric oxide is markedly enhanced in neonatal microglia (34, 36). Thus, it is possible that glia cells have different effects on acute brain ischemic lesions in neonatal and adult mice due to their different immune response abilities.

Apart from mouse age, the difference in mouse gender and genetic background may also affect the outcome of HI mice. For example, there is a gender difference in HI-induced acute brain injury in C57BL6 mice (26), as female C57BL6 mice have smaller brain lesions than male mice after HI induction. It is thought that different apoptotic cell death mechanisms are activated in neonatal male and female brains after HI insult (37). A recent tamoxifen-induced conditional genetic microglia depletion study by also showed aggravated brain injury after cerebral HI in neonatal mice and more predominantly in males (27). In the current study, we did not find a gender difference in brain lesion at 48 h after HI in mice treated with PLX3397 or the vehicle. One reason is that gender differences may not be statistically significant at 48 h after HI, as a previous study indicated that female HI mice have smaller brain lesions than male mice at 72 h but not earlier than 24 h after HI induction (26). Another reason is that we used CD1 mice in this study and this strain is more susceptible to HI injury than C57BL6 mouse pups (22). The difference in genetic background may affect the brain pathology development including microglia activity and neural function after HI, resulting in different brain injury mechanisms and outcomes. In addition, not all analysis had equal sex distribution in our results, such as the brain lesions and the cell death between mice treated with PLX3397 and vehicle as shown in Figures 2B, 5. Thus, the potential effects of sex difference on HI induced brain lesion and cell death between mice treated with PLX3397 and vehicle needs to be further studied. In our further studies, we will pay attention to whether the gender, mouse strain, and even HI model procedure affect microglia activity and HI outcomes by PLX3397 in neonatal mice.

In this study, PLX3397 altered the immune profile in the brain of HI mice, manifested in the decrease of microglia, brain infiltrated immune cells, and chemokine/cytokine expression in brain tissue. Although we did not study the downstream molecular mechanism of PLX3397 on brain lesion and neuron death, microglia related neuroinflammation may be a potential mechanism. Studies suggest that microglia are rapidly activated by acute brain injuries including brain ischemia and contribute to acute brain injury and chronic brain repair (38). As for HI, microglia exhibit different functional phenotypes during injury and brain repair, similar to M1, M2A, and M2b phenotypes proposed in other models (12, 39). Microglia mainly differentiate into the M1 phenotype and are thought to play a detrimental role in brain injury after HI, at least in the acute stage (40–43), though the mechanism is not fully understood. Thus, the effects of PLX3397 on brain lesion and neuron death may be related to inhibited M1 polarization and brain inflammation in this study. Furthermore, our cytokine/chemokine array result provides additional cues of the mechanism on attenuated brain injury in HI + PLX3397 mice. Among the 18 differently expressed cytokines/ chemokines, some of them may be directly harmful to neurons. For example, a recent study showed that CCL6 can aggravate hypoxia-reoxygenation-induced cell apoptosis (44). In our study, we found that protein and mRNA levels of CCL6 were significantly reduced in whole brain tissue of HI mice treated with PLX3397. This reduced CCL6 level may be related to neuron protection in HI mice treated with PLX3397. Another example is M-CSF. It is reported that astrocytes constitutively express M-CSF that increases phagocytosis and proliferation of microglia (45, 46). M-CSF also promotes the differentiation of monocytes into M2 macrophages with an “anti-inflammatory” cytokine repertoire and functions (47, 48). It is interesting that mice treated with PLX3397 had a higher protein level of M-CSF after HI induction in our study. This high level of M-CSF may facilitate the clearance of dead cells and subsequently brain repair. Thus, it is worth dissecting the potential mechanism of PLX3397 on brain injury in HI mice in future studies.

Overall, this study demonstrates that inhibition of CSF1R suppresses neuroinflammation and HI injury in the neonatal brain.

## Data Availability Statement

The raw data supporting the conclusions of this article will be made available by the authors, without undue reservation.

## Ethics Statement

The animal study was reviewed and approved by the Committee on the Ethics of Animal Experiments of Tianjin Neurological Institute (Tianjin, China).

## Author Contributions

XZ, SZ, and CZ formulated the study concept and designed research. BZ, YR, SW, FZ, and HH performed experiments. BZ, YR, and SW analyzed the data, interpreted the results, and wrote the paper. All authors contributed to the article and approved the submitted version.

## Conflict of Interest

The authors declare that the research was conducted in the absence of any commercial or financial relationships that could be construed as a potential conflict of interest.
